# A Visualization Technique of a Unique pH Distribution around an Ion Depletion Zone in a Microchannel by Using a Dual-Excitation Ratiometric Method

**DOI:** 10.3390/mi9040167

**Published:** 2018-04-02

**Authors:** Katsuo Mogi

**Affiliations:** Molecular Profiling Research Center for Drug Discovery (Molprof), National Institute of Advanced Industrial Science and Technology (AIST), 2-4-7 Aomi, Koto-ku, Tokyo 135-0064, Japan; mogi.k@aist.go.jp; Tel.: +81-3-3599-8251

**Keywords:** ion concentration polarization, ion depletion zone, Nafion, microfluidic device, pH indicator, fluorescein isothiocyanate (FITC)

## Abstract

The ion depletion zone of ion concentration polarization has a strong potential to act as an immaterial barrier, separating delicate submicron substances, including biomolecules, without causing physical damage. However, the detailed mechanisms of the barrier effect remain incompletely understood because it is difficult to visualize the linked behavior of protons, cations, anions, and charged molecules in the thin ion depletion zone. In this study, pH distribution in an ion depletion zone was measured to estimate the role of proton behavior. This was done in order to use it as a tool with good controllability for biomolecule handling in the future. As a result, a unique pH peak was observed at several micrometers distance from the microchannel wall. The position of the peak appeared to be in agreement with the boundary of the ion depletion zone. From this agreement, it is expected that the pH peak has a causal connection to the barrier effect of the ion depletion zone.

## 1. Introduction

Understanding the ionic characteristics of molecules in solution is a useful tool for their handling, such as in concentration and separation. This is important in the fields of molecular chemistry and molecular biology, among others [1,2,3,4].

Ion concentration polarization (ICP), which can be easily used by applying voltage to a solution across an ion-exchange membrane, is a well-known and convenient concentration technique for dialysis [5,6,7]. Recently, not only this concentration technique, but another technique based on an almost negligible phenomenon called ion depletion, which occurs near a membrane under steady-state ICP, have attracted attention [8,9]. The microscale ion depletion zone has a peculiar ability to repel charged particles, including biomolecules. The strong potential of using this effect to provide an immaterial barrier for the separation of delicate submicron substances, without causing physical damage, has been reported [10,11,12]. The microscale barrier can be effectively employed by building an ICP system into a microfluidic device, in which a microchannel provides sufficient space for using the valid range of the barrier [13].

It is generally believed that the microscale barrier effect of the ion depletion zone is caused by nanoscale ionic behavior. This is due to the fluidic and electrochemical forces around the ion-exchange membrane [9]. Hence, it is important to investigate pH distribution in nanochannels and porous materials [14,15,16,17]. However, the detailed mechanism of the barrier effect remains incompletely understood because it is difficult to visualize the linked behavior of protons, cations, anions, and charged molecules in the thin ion depletion zone.

In this study, to make better use of the barrier effect by controlling the volume of ion transfer, including protons, the proton concentration distribution in the ion depletion zone was verified to clarify the role of proton behavior in the barrier effect. For verification, the pH was measured by the dual excitation ratio method using fluorescein isothiocyanate (FITC), one of the most common negatively charged fluorophores used as a pH indicator [18,19]. Furthermore, the boundary of the barrier of the ion depletion zone was estimated from the concentration distribution of the FITC molecule, which was subjected to a repulsive force.

## 2. Materials and Methods

### 2.1. Ion Concentration Polarization (ICP) in Microchannels

**Figure 1:**
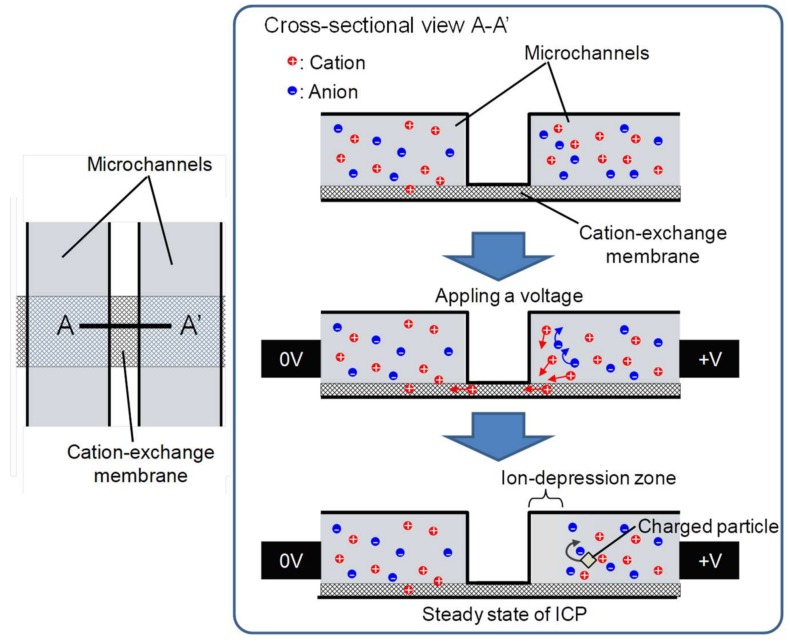
Schematic of microchannels for ion concentration polarization (ICP). The left schematic is a top view of a region composed of two microchannels and a cation-exchange membrane. The right schematic is a cross-sectional view of A-A’ in the left schematic. Generating a difference in potential causes the movement of cations through the membrane, whereas anions remain in their original channels. Under steady-state ICP, an ion depletion zone is formed around the high-potential side of the membrane.

To make effective use of the almost negligible phenomena in an ion depletion zone, an ICP system was scaled down using a microfluidic device [20,21]. Figure 1 shows an illustration of two microchannels in the microfluidic device. These two microchannels were connected by a cation-exchange membrane. The cross-sectional view of A-A’ shows the movement of cations and anions in the channels. When voltage was applied to generate a difference in potential between the two channels, with the right channel at a higher potential than the left, cations moved through the membrane from right to left and anions remained in their original channels. This formed an ion depletion zone around the right side of the membrane under steady-state ICP, and this ion depletion zone repelled all charged particles. While the ion depletion zone expanded due to the potential difference, the dimension of the zone was maintained by ions which were continuously supplied by constant flow.

### 2.2. Measurement Principles

To visualize proton behavior as an indicator of ion balance, the pH was measured around the ion depletion zone. The pH in the microchannel was non-invasively measured by a dual-excitation ratiometric technique using FITC [22]. In a microchannel with a short light path for observation, the fluorescent intensity of FITC, $I_{f}$, excited by excitation beam of intensity $I_{e}$ is given by Equation (1):

(1) $$I_{f}\left(\mathrm{pH},\;C\right)\;=\;I_{e}C\phi_{e}\varepsilon_{e}\;\left(\mathrm{pH}\right)\;-\;I_{back,e}\left(\mathrm{pH},\;C\right)$$

In Equation (1), e (nm) is the wavelength of the beam, C (kg/m^2^) is the FITC concentration, $\phi_{e}$ is the quantum efficiency, $\varepsilon_{e}$ (m^2^/kg) is the molar absorption coefficient, and $I_{back,e}$ is the background intensity. Hence, the normalized fluorescent intensity depends only on the pH, as given by Equation (2):

(2) $$I_{f}\left(\mathrm{pH}\right)\;=\;\frac{I_{488}\phi_{488}\varepsilon_{488}\;\left(\mathrm{pH}\right)-I_{back,488}\left(\mathrm{pH}\right)}{I_{458}\phi_{458}\varepsilon_{458}\left(\mathrm{pH}\right)-I_{back,458}\left(\mathrm{pH}\right)}$$

### 2.3. Standard Solutions for pH Calibration

Standard solutions for pH calibration containing 9 × 10^−6^ M FITC were adjusted to pH 2.05 and 2.68 with sodium dihydrogenphosphate dihydrate and phosphoric acid, to pH 4.61 with potassium dihydrogenphosphate, to pH 5.74, 6.52, and 7.28 with potassium dihydrogenphosphate and disodium hydrogen phosphate 12-water, to pH 6.76 with sodium dihydrogenphosphate dehydrate and disodium hydrogen phosphate 12-water, and to pH 8.18 with potassium dihydrogenphosphate and potassium hydrate.

## 3. Experimental Section

### 3.1. Experimental Setup

**Figure 2:**
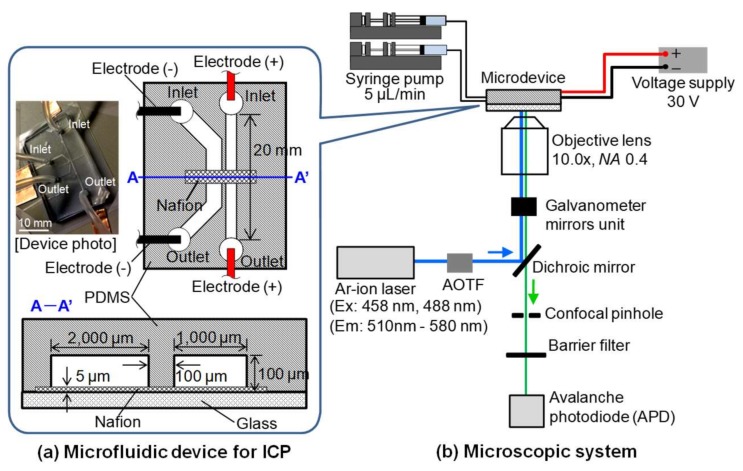
Schematic of the experimental system. (**a**) Microfluidic device for ICP made of glass and substrates; (**b**) A schematic of the microscopic system used for pH measurement. Excitation beams at 458 nm and 488 nm were used in a dual-excitation ratiometric method. The microfluidic device on the microscopic system was connected to a syringe pump and a voltage supply.

The microfluidic ICP device was composed of a glass substrate with a cation-exchange membrane pattern and a polydimethylsiloxane (PDMS) substrate with two microchannels, as shown in Figure 2a. One of the channels, which was 1 mm wide and 100 µm high, was used for the injection of FITC solutions and was called the “main channel”. The other channel, used for the injection of distilled water, was named the “Ground (GND) channel” and was 2 mm wide and 100 µm high. The central areas of the microchannels were connected with a Nafion (DuPont) membrane, which is a band-shaped pattern with a width of 100 μm across the channels. The FITC solution in the main channel and the distilled water in the GND channel were drawn in at 5 µL/min by a syringe pump (KDS 200 syringe pump, KD Scientific Inc., Holliston, MA, USA). Then, ion transfer for ICP around the Nafion pattern in the main channel was generated by applying a voltage of 30 V. A voltage supply (P4K-80M, Matsusada Precision Inc., Bohemia, NY, USA) was connected to the electrode on the inlet and outlet ports of the microchannels. 

The fluorescent intensity of FITC in a microchannel was measured with a confocal laser scanning microscope system (TCS STED-CW; Leica Microsystems, Leica Microsystems Inc., Buffalo Grove, IL, USA), as shown in Figure 2b [23]. Excitation beams at 458 nm and 488 nm were selected from an Ar-ion laser source using an acousto-optic tunable filter (AOTF). A 1.55 mm^2^ field of the main channel around the Nafion was scanned with the excitation beam, which was controlled by galvanometer mirrors. The FITC fluorescence was obtained by avalanche photodiode as a 16-bit intensity image.

### 3.2. Device Fabrication

**Figure 3:**
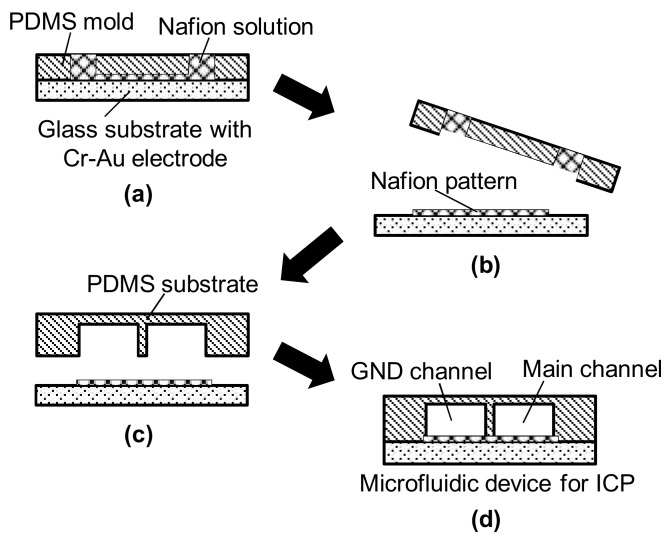
Schematic of the fabrication process for Nafion patterning. (**a**) Cross-sectional view of Nafion patterned on a glass substrate using a polydimethylsiloxane (PDMS) mold; (**b**) Nafion pattern cured at 100 °C for 10 min after peeling off the mold; (**c**) Assembly of the PDMS substrate onto the glass substrate; (**d**) Completed microfluidic ICP device.

The microchannels were fabricated on PDMS using a commonly-employed soft lithography technique [24,25,26]. A band-shaped cation-exchange membrane was patterned on a glass substrate using a Nafion solution (Nafion® 20 wt % dispersion, DuPont). The shape of the Nafion solution was formed using a PDMS mold with a microchannel 100 µm wide and 25 µm high. Uncured Nafion solution was injected into the channel of the PDMS mold on the glass substrate, as shown in Figure 3. Then, the substrate with the PDMS mold containing the Nafion solution was cured at 100 °C for 10 min. After peeling off the mold, the Nafion pattern was formed on the glass substrate. The Nafion-patterned glass substrate was aligned with the microchannel substrate made of PDMS to complete the microfluidic ICP device.

## 4. Results and Discussion

**Figure 4:**
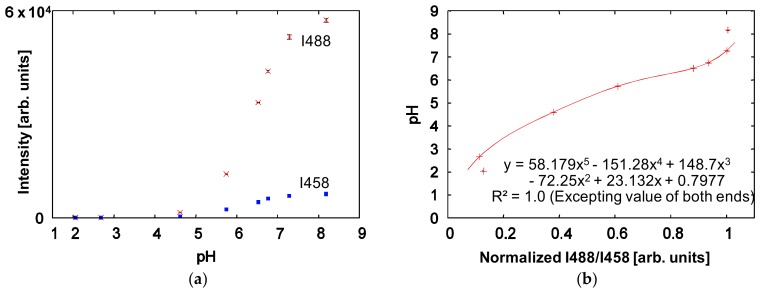
Calibration curve between pH value and fluorescein isothiocyanate (FITC) intensity. (**a**) FITC intensities of pH standard solutions obtained by dual excitation at 458 nm and 488 nm. Each intensity shown is the mean value over a 28 × 28 µm^2^ square, and the error bar shows the standard deviation; (**b**) A calibration curve between pH value and the quotient of the intensities, normalized by the maximum value. The calibration curve is a polynomial approximation: $y=58.2x^{5}-151.3x^{4}+148.7x^{3}-72.3x^{2}+23.1x+0.8$. The R-squared value was 1.0 from a pH of 2.68 to a pH of 7.28, excluding the endpoints of 2.05 and 8.18.

To generate a calibration curve of pH vs. the experimental value, fluorescent intensities obtained from pH standard solutions with pH values of 2.05, 2.68, 4.61, 5.74, 6.52, 6.76, 7.28, and 8.18 were measured by dual excitation at 458 nm and 488 nm, as shown in Figure 4a. Each intensity value in the figure is the mean value over the scanned area (28 × 28 µm^2^ square) at the center of the microchannel, and the error bar shows the standard deviation of the pixel data. From the intensity values in Figure 4a, the quotient of the intensities represented by Equation (2), normalized by the maximum value, was obtained as shown in Figure 4b. The calibration curve in Figure 4b was generated by a polynomial approximation, and the R-squared value was 1.0 in the range from pH = 2.68 to pH = 7.28. Hence, it was decided that the calibration curve is useful to estimate the pH distribution in ranges other than pH = 2.05 and pH = 8.18. Therefore, it was decided that this calibration curve would be applied to the estimation of the pH distribution in that range.

**Figure 5:**
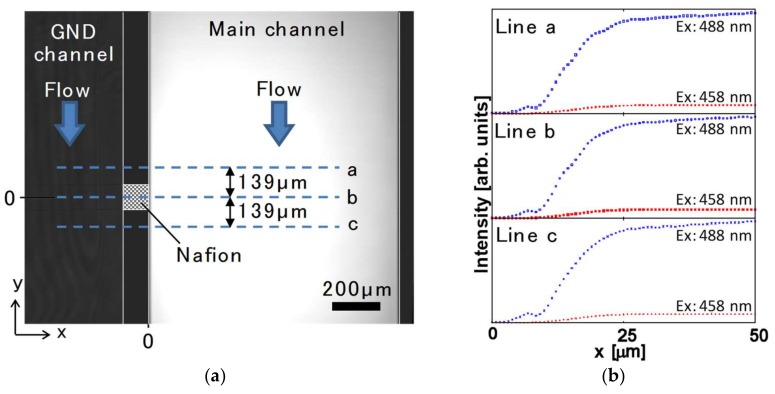
Measurement area and the FITC intensity distribution. (**a**) Photo of fluorescent intensity of FITC in steady state at the ion depletion zone in the microchannel. The zero position of the *x*-axis is the wall surface of the main channel, and the zero position of the *y*-axis is the center of the Nafion pattern. Lines a, b and c are parallel to the *x*-axis, and their positions on the *y*-axis are *y* = 139 µm, 0 µm and −139 µm; (**b**) Intensity distributions obtained by dual excitation at 458 nm and 488 nm under a steady-state ion depletion zone at lines a, b and c.

To estimate the pH distribution in the ion depletion zone based on the calibration curve, an image of the fluorescent intensity of FITC around the Nafion pattern was captured, as shown in Figure 5a. The zero position of the *x*-axis was defined as the wall surface of the main channel, and the zero position of the *y*-axis was defined as the center of the Nafion pattern. Figure 5b shows an intensity distribution in the steady stage at lines a, b and c in Figure 5a. The lines are parallel with the *x*-axis, and the positions on the *y*-axis are *y* = 139 µm, 0 µm and −139 µm. The effect of the autofluorescence of the Nafion was removed by normalization using the background intensity of the channel without Nafion.

**Figure 6:**
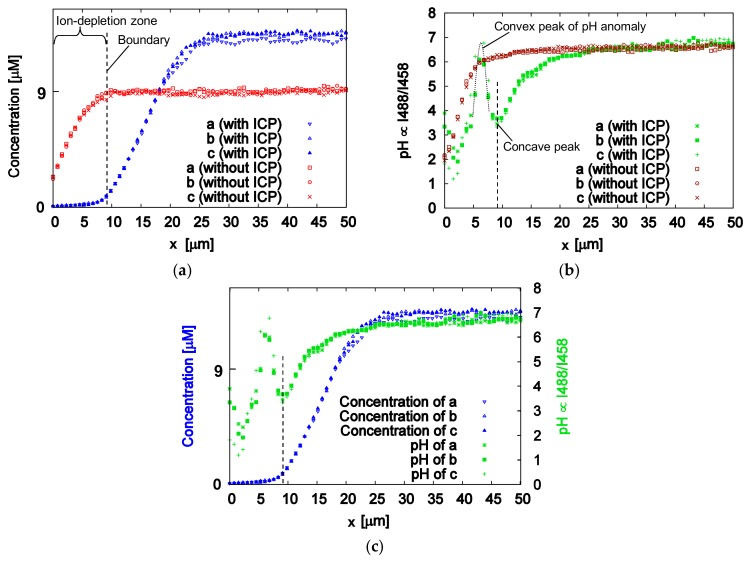
FITC concentration distribution and pH distribution at the ion depletion zone generated by ICP. (**a**) FITC concentration distributions under steady state at lines a, b and c while applying ICP, with ICP and without ICP. The dotted line at *x* = 9.0 ± 0.10 µm is the boundary of the ion depletion zone; (**b**) pH distribution obtained from the intensity quotient values after excitation at 458 nm and 488 nm (“I488/I458”), with ICP and without ICP. The pH distribution with ICP has a convex peak at *x* = 6.3 ± 0.15 µm and a concave peak at *x* = 9.1 ± 0.08 µm; (**c**) FITC concentration and pH distributions at lines a, b, and c under steady-state ICP.

Figure 6a shows the FITC concentration distributions at the lines a, b and c in Figure 5a, as obtained from the fluorescent intensity excited by the 458 nm laser. As seen in the figure, the rise curve of the concentration drifted from the microchannel wall to the center of the channel by the repulsive force of the ion depletion zone, and the top of the concentration curve became 1.5 times higher from the force. In this study, the rising point (*x* = 9.0 ± 0.10 µm) of the drifted curve, which was the *x*-intercept of the approximate line between *x* = 11 µm and 19 µm, was defined as the boundary of the ion depletion zone.

On the other hand, Figure 6b shows the value of the pH distribution (“I488/I458”) obtained from the intensity quotient values for excitation at 458 nm and 488 nm. Scrupulous attention is required to treat the pH values, because the values lower than pH 2.68 and higher than pH 7.28 were out of range in application of the calibration curve. As illustrated by the dotted line in Figure 6b, a convex peak appeared while applying ICP. The position coordinate of the peak is *x* = 6.3 ± 0.15 µm, which was obtained by Gaussian fitting. Although measurement error is considered to be included due to the low concentration of FITC at the right of the convex peak, a slight increase in pH due to a decrease in proton by passing through the Nafion pattern was observed at the closest side of the microchannel wall. Interestingly, it can be clearly seen that the bottom of the concave peak (*x* = 9.1 ± 0.08 µm) to the right of the convex peak corresponded well with the boundary of the ion depletion zone, as shown in Figure 6c. It may be suspected that the sharp increase and decrease in pH is a factor in forming a specific electrochemical equilibrium state that generates the boundary of an ion depletion zone, which acts as a barrier to keep charged substances away.

## 5. Conclusions

The pH distribution around an ion depletion zone in a microchannel was measured by a dual excitation ratio method with FITC to estimate proton behavior. In a microchannel of PDMS without ICP, pH is slightly decreased near the microchannel wall due to the electric double layer. On the other hand, a unique pH peak, which has never been previously reported, was observed at *x* = 6.3 ± 0.15 µm from the microchannel wall in cases with ICP. Furthermore, the position of the unique peak was in agreement with the boundary of the ion depletion zone, which was estimated from the rising point of the FITC concentration. This agreement indicates that the barrier effect of the ion depletion zone has a profound causal connection with the pH anomaly. Although there has not yet been enough investigation to clarify the phenomena related to ionic behavior in the ion depletion zone, it can be expected that this report may play an important role in better utilizing barrier effects with high controllability for biomolecule handling in the future.
